# “PADding” My Career with Dr. Mary Ellen Avery

**DOI:** 10.3389/fped.2014.00029

**Published:** 2014-04-07

**Authors:** Heber C. Nielsen

**Affiliations:** ^1^Department of Pediatrics, Tufts Medical Center, Boston, MA, USA

**Keywords:** mentoring, Mary Ellen Avery, neonatology, tribute, memorial

On Wednesdays at 2:00 p.m., faculty, fellows, and research associates in the Joint Program in Neonatology (JPN) at Harvard would meet in the conference room of the Seeley Mudd building for Research Conference. Dr. Avery would come bustling in, settle in a chair, and ask the group “So, what exciting discoveries have you made this week?” Those Wednesday conferences with Dr. Avery became one of the major mentoring environments of my neonatal fellowship training. I now group the many opportunities to gain mentoring from Dr. Avery into three main categories, which I like to think of as Prepare, Ask, and Discover (PAD).

Very early in my first year of training, I conceived a research question involving perinatal/neonatal hypoxia and pulmonary hypertension. I thoroughly studied the literature to educate myself on what was known about oxygenation and pulmonary blood pressure, and created a research hypothesis and approach. Soon it was my turn to present a proposed fellowship research project to the Research Conference. I discussed the background to my idea, the science it was based on, my hypothesis, and my proposed approach. At the end there were, of course, many questions. After all, I was just a first year fellow and needed to learn my place. The discussion was capped off with Dr. Avery’s comment “there is a huge literature in adults on hypoxia and pulmonary hypertension; I think you need to go back to the library and study that to better develop your rationale, hypothesis, and study approach.” I knew about that literature; I had studied it carefully and it had definitely influenced the project I had so carefully designed and presented. I was strongly tempted to say “yes, I know, I read all of that,” fortunately, I did not. As I thought about that humbling comment from Dr. Avery over time I realized there was a significant learning insight for my career. If I cannot show that I am intimately familiar with all the relevant literature as I present a research idea or study, then I cannot expect to get others interested and excited about the project. This truth has had a major impact on the development of my skills in presentations and writing of papers and grants. “Prepare” is a necessary component for success in our academic life.

We frequently had outside speakers at these Wednesday conferences, including speakers on topics far afield of the research interests and even expertise of the group. Dr. Avery was always attentive. What impressed me was that she was always there with questions. It didn’t matter if the questions were clever or if they were simply way off the mark. She didn’t hesitate to state or even show that she simply was not informed on the topic. A few times she asked a question that caused private smiles, because it seemed that she had overlooked some of her basic biology in posing the question. But she was never embarrassed. As time went on I noted that by asking questions, no matter how basic or uninformed on the subject, her personal knowledge of that subject grew such that in the future she was able to converse knowledgeably on the topic. This was the second major learning insight for me. “Ask” questions; no one should be embarrassed by a lack of even basic knowledge on a subject. Don’t worry about the possibility of coming across as uneducated. “Ask” is the major way we have to learn about things. This component of Dr. Avery’s mentoring is one I have had to continue to work on throughout my career.

Let me come back to Dr. Avery’s signature question “What exciting discoveries have you made this week?” For a while this seemed odd. How could she expect that new discoveries would be made each week? Obviously, research is time-intensive; new discoveries don’t just pop up each day! But with time I saw that she was teaching us two important attitudes. First, our research is exciting and we should always approach it that way. Second, every finding, no matter how small, is new and unique, and deserves to be celebrated. We don’t have to wait until a research project is finished to derive the joy of scientific learning and discovery. Thus, “Discover” is an element of every day in the lab. Without it we just aren’t approaching our work with the right attitude.

Prepare, Ask, and Discover was central for Dr. Avery. It governed our relationships with her, it stimulated her interest in following us in our careers after we left the incubator, and it led to some of the most imaginative thinking I have witnessed. For example, in a Wednesday conference Dr. Avery proposed the concept that male and female cells from non-reproductive organs have fundamental differences in their biology. This concept has only recently become a major consideration in human translational biology research. In 1980, Dr. Avery told us to begin to look out for unusual cases of neonatal respiratory distress syndrome, because we would begin to find mutations in surfactant protein genes that underlie some cases. This preceded the identification of such mutations by some 20 years. In retrospect, it was obvious, but in 1980, it was a new and far sighted prediction.

One final tribute. As a mentor I am only as good as the people who mentored me. Dr. Avery has had a profound impact on my career and I hope on those who have developed under my guidance. The impact and legacy of a great physician–scientist like Dr. Avery will go on and on through the generations of medical science.

